# *The Organon* and Materia Medica Club of the Bay Cities of California

**Published:** 1896-05

**Authors:** W. E. Ledyard

**Affiliations:** Secretary


W. E. LEDYARD, *Secretary.*

Reported by Eleanor F. Martin, M. D.

---

### *THE ORGANON* AND MATERIA MEDICA CLUB OF THE BAY CITIES OF CALIFORNIA.

The regular semi-monthly meeting was held at the office of Dr. George H. Martin, 606 Sutter Street, San Francisco, December 20th, 1895.

Members present: Drs. J. M. Selfridge, S. E. Chapman, W. E. Ledyard, G. J. Augur, M. F. Underwood, M. T.

Wilson, A. McNeil, C. M. Selfridge, and George H. Martin. Visitors: Drs. A. J. Forget and A. N. Couture and Mr. Huffman.

The meeting was called to order at 8.15 P. M. by the President, Dr. J. M. Selfridge. The minutes of the previous meeting were read by the Secretary, and after a slight correction by Dr. McNeil were approved.

Dr. Chapman presented a patient of his to the members, wishing their opinion as regards diagnosis, etc. Mr. E., age, sixty-three, rancher, met with an accident last May, almost immediately after which the following stomach symptoms appeared: excessively acid condition of the stomach; flatulency; empty eructations, which sometimes relieves; constant distress in stomach; feels very badly when stomach is empty and seems to feel somewhat better by eating; not much nausea, and very little vomiting; constantly smelling unpleasant odors, better out in the open air; smelling of cooking food particularly disagreeable. The patient's history showed an attack of chills and fever dating back forty years, with some stomach trouble of about thirty years' standing, but which had not been very severe until the accident in May. Dr. Chapman stated that he had met several cases similar to this one, and that he was of the opinion that this case was carcinomatous, and, if so, would like to prove that homœopathic remedies would cure these troubles.

Dr. J. M. Selfridge—In a man of this patient's age, with the symptoms of long standing, I should be inclined to think it something malignant, though this opinion might be modified by further investigation. I had one case of a man who had suffered with indigestion for years, but finally the symptoms started in with great severity. Degeneration commenced in the pancreas, extended to the stomach, and killed him.

Dr. Ledyard—I think there was a suppression of the chills in Dr. Chapman's case, and I think this fact should be kept in mind during the treatment.

Dr. Chapman—I have thought of *Lycopodium* or *Carbo-vegetabilis* as suitable remedies for this case.

Dr. Underwood (on learning that the patient had also had a suppression of gonorrhœa)—I saw such a case cured by bringing out the suppressed disease. I would suggest *Medorrhinum* to bring out the suppressed discharge in this case.

The President then proceeded with the regular business of the Club, suggesting, first, that the members select a special place to meet in San Francisco, so that the members will always know just where the meetings are to be held; whereupon Dr. Martin offered his offices to be used for the purpose. Dr. Wilson moved that the Club accept Dr. Martin's offer with thanks, and that hereafter the San Francisco meetings be held at Dr. Martin's offices. This was seconded by Dr. Ledyard. Carried.

Dr. McNeil then introduced Dr. A. N. Couture and Dr. J. M. Selfridge introduced Dr. A. J. Forget. Upon motion, these gentlemen were extended the courtesies of the Club with privilege of the floor.

The President then appointed Dr. Martin reader of the evening, who commenced *The Organon* at Section 19, continuing to 22.

DISCUSSION.

Dr. Ledyard—I had a letter from a lady who suffers from asthma. She writes to ask my advice, as she expresses it, regarding a preparation which she has heard will cure asthma. The preparation contains Iodide of Potash, Bromide of Potash, and Kola-nut. I sent her the symptoms produced by Iodide and Bromide of Potash, but the Kola-nut I knew nothing about. She said many cases had been cured by this preparation. How could these drugs cure asthma? They each produce different symptoms.

Dr. J. M. Selfridge—Perhaps the combination formed a new remedy, and in this way made a cure.

Dr. McNeil—The Kola-nut is evidently one of the most powerful stimulants. It is like Coca. Alcohol is not to be compared to these drugs as stimulants. Under their influence men can make wonderful exertions, but their secondary reactions are, in consequence, extremely depressing. In crossing over the Andes the Indians will carry heavy burdens for days without food, but under the effects of these stimulants. At the age of thirty, however, they are more like walking mummies than men.

Dr. J. M. Selfridge—We do not know by what this lady wrote that any of the patients were cured. What she would call a cure and what we would call a cure might be a very different thing.

Dr. Underwood—I have a patient now who has used it for years, and she gets relief, but it does not cure. The allopaths call palliation cure.

Dr. J. M. Selfridge—I know of many cases which have been relieved by the removal of the so-called third tonsil, and some call such cases cures. I think it is too early yet to be sure of a cure by the removal of the third tonsil.

Dr. McNeil—In a case of horrible eczema, the patient could not even wear slippers, as the feet were in such a bad condition. Had tried all physicians, but did not seem to get well. Finally *used Cuticura Soap, and there was a most remarkable cure.* I think many of these cures are accidental, and it is a mistake to think that because a certain thing cures in one case that it will do so in every case.

Dr. Chapman—You take the ground, then, that all cures are homœopathic?

Dr. McNeil—Yes. We are the only school that recognizes the difference between individuals.

Dr. Couture—Does it necessarily follow that the asthma would come back again, or may it not come back again in the form of some other disease?

Dr. J. M. Selfridge—As in *cure* by the removal of the third tonsil. They are apt to be like the Frenchman's operation: the operation was a success, but the patient died.

Dr. Ledyard—In a case like that, would it not be a suppression of a suppression, which one would think would be almost impossible to cure?

Dr. Chapman—It strikes me that a great many of our prescribers will ignore the totality of symptoms to prescribe for an old itch, etc. I think *we are bound to prescribe for the totality of symptoms,* no matter what has been suppressed.

Dr. Ledyard—That is right; but if we prescribe for the totality of the symptoms, and yet the condition does not pass away, I then think it best to give the nosode to bring out the suppressed disease.

Dr. Underwood—I do not give the nosode homœopathically. It is given to bring back the suppressed disease, that I may cure it with the indicated remedy.

Dr. McNeil—I am a good deal like Dr. Chapman on that point. The question of nosodes has been made too much of. Anything that cannot come up straight and fair with *similia similibus curantur*, sooner or later will come to grief. The psoric theory does not violate the law of similars; but if you have a case of psora and give Psorium simply for that reason, it would be given against the law, and would, sooner or later, be a failure. If *Tuberculinum*, *Syphilinum*, or any other nosode, is given according to the law of cure, it will cure; but it will not do so in any other way. Homœopathy does not consist simply in giving a high potency, bu *the totality of symptoms must always be met.*

Sections 23–27 were next read.

### Discussion.

Dr. McNeil—There has been a gross misconception as to the meaning of one point here. A number of years ago a man made an assertion in the *American Homœopath* that Hahnemann did not practice with the high potencies; and that all who used high potencies were not following his teachings. This man said that Homœopathy had no right to the high potencies. The point is this: that a *drug which is more potent than the disease should be used.* If any one will read that as seeking the truth, and what follows, he will see that the 30th potency that Hahnemann used was not potent enough to overcome this. I quoted from the *Materia Medica Pura*, and the *Chronic Diseases* and sent the *American Homœopath* an answer to what this man wrote, but they never published it.

Section 28 was read.

DISCUSSION.

Dr. J. M. Selfridge—I have always thought that Hahnemann made a mistake in explaining the effect of drugs.

Dr. McNeil—Hahnemann meant that he was satisfied with what he found out about the effects of drugs, and we can take his experience or not as we wish.

Section 29 was next read.

DISCUSSION.

Dr. J. M. Selfridge—It seems to me that the cure of disease is better explained by recent and more scientific explanations. *Sickness is disturbed molecular action* instead of deranged "vital force."

Dr. Underwood—I would like to know what it is that causes the molecules to be disturbed.

Dr. McNeil—Talking of disturbed molecular motion is simply getting a scientific nomenclature for *deranged vital force.* I think the thing itself remains.

Dr. Chapman—I believe that *it is the spiritual man that is affected.*

Dr. McNeil—How do you account for the spiritual sickness in animals?

Dr. Chapman—We may be able to account for that too. This talking of metaphysics is talking about something you do not understand, and what no one can understand.

Dr. J. M. Selfridge—I have no idea of disputing Hahnemann, but I do not think his explanation is the best.

Dr. McNeil—Dr. Selfridge's explanation is more modern and scientific perhaps, but it is still the same as Hahnemann's. There is one point to be remembered: At the time that Hahnemann lived, the reign of law had not lived. He introduced it in the law of medicine. Every intelligent man believes now in the reign of law. An intelligent allopath will tell you that he believes in almost every law but that of medicine. Hahnemann shows that a man cannot get out of the reach of law by being sick.

The President then declared the meeting adjourned, to meet again the first Friday in January, at the office of Dr. George H. Martin, 606 Sutter Street, San Francisco, when the reading of *The Organon* would be commenced at Section 29.

